# Gonadal function in male patients after treatment for malignant lymphomas, with emphasis on chemotherapy

**DOI:** 10.1038/sj.bjc.6604892

**Published:** 2009-01-20

**Authors:** C E Kiserud, A Fosså, T Bjøro, H Holte, M Cvancarova, S D Fosså

**Affiliations:** 1 Department of Clinical Cancer Research, National Resource Center for long term effects after Cancer, Norwegian Radium Hospital, Rikshospitalet University Hospital, Oslo 0310, Norway; 2 Department of Oncology, Cancer Clinic, Norwegian Radium Hospital, Rikshospitalet University Hospital, Oslo 0310, Norway; 3 Department of Medical Biochemistry, Rikshospitalet University Hospital and University of Oslo, Oslo, Norway; 4 Faculty Division the Norwegian Radium Hospital, University of Oslo, Oslo O316, Norway

**Keywords:** male lymphoma survivors, chemotherapy, gonadal function

## Abstract

Gonadal function was assessed in male lymphoma survivors based on serum hormone levels (LH, FSH, testosterone, SHBG), and was related to treatment, age and observation time. Male patients ⩽50 years at diagnosis treated for Hodgkin's (HL) and/or non-Hodgkin's lymphoma (NHL) at the Norwegian Radium Hospital from 1 January 1980 to 31 December 2002 were included. Five treatment groups were defined: 1: radiotherapy only and/or low gonadotoxic chemotherapy (both HL and NHL)(‘No/low’), 2: medium gonadotoxicity chemotherapy for NHL (‘med-NHL’), 3: medium gonadotoxicity chemotherapy for HL (‘med-HL’), 4: highly gonadotoxic chemotherapy for NHL (‘high-NHL’), 5: highly gonadotoxic chemotherapy for HL (‘high-HL’). Gonadal hormone levels were categorised into three groups: 1: All gonadal hormones within normal range (normal), 2: Isolated elevated FSH, with LH, SHBG and testosterone within normal range (exocrine hypogonadism), 3: Testosterone below and/or LH above normal range (endocrine hypogonadism). One hundred and forty-four (49%) of the patients had normal gonadal hormones, 60 (20%) displayed exocrine hypogonadism and almost one-third (*n*=90, 30%) had endocrine hypogonadism. Compared to those treated with no/low gonadotoxic chemotherapy patients from all other treatment groups had significantly elevated risk for exocrine hypogonadism. Patients from the other treatment groups, except those in the med-NHL group, also had significantly elevated risk for endocrine hypogonadism compared with the group treated with no/low gonadotoxic chemotherapy. Men aged above 50 years at survey were about five times more likely to have endocrine hypogonadism compared with those less than 40 years. Because of the adverse health effects following long-lasting endocrine hypogonadism, gonadal hormones should be assessed regularly in male lymphoma survivors, especially after treatment with alkylating agents and high-dose chemotherapy with autologous stem cell support and in male patients who are 50 years and older.

Testicular dysfunction can be a late side effect after cancer treatment. It is known that post-treatment spermatogenesis in male survivors after malignant lymphoma is dependent on the type of chemotherapy, the drugs’ cumulative dose, the radiation doses to the testicles from scattered or direct irradiation and the time since last treatment (Lee *et al*, 2006).

Further, some studies have shown that pre-treatment spermatogenesis in survivors after Hodgkin's lymphoma (HLSs) is impaired at a greater degree than in survivors after non-Hodgkin's lymphoma (NHLSs) (Botchan *et al*, 1997; Fitoussi *et al*, 2000; Rueffer *et al*, 2001). Post-treatment gonadal dysfunction may thus be associated with pre-treatment fertility problems.

During recent years evidence has emerged that malignant lymphoma survivors may have subnormal serum levels of testosterone and/or elevated LH (Whitehead *et al*, 1982; Viviani *et al*, 1991; Howell *et al*, 1999) However, there are a few large studies confirming these findings, and even fewer studies are published which compare HLSs with NHLSs. On the other hand, endocrine hypogonadism may increase the risk of severe health problems such as premature osteoporosis and insulin-resistance (Finkelstein *et al*, 1987; Howell *et al*, 2000; Greenfield *et al*, 2007).

In adult men the testicles have both endocrine (hormone production) and exocrine (spermatogenesis) function, controlled by the hypothalamus – pituitary – gonad axis. Gonadotropin-releasing hormone (GnRH) from the hypothalamus promotes the production of the luteinizing hormone (LH) and follicle-stimulating hormone (FSH) from the anterior pituitary. Simplified, LH stimulates the Leydig cells to produce testosterone; FSH and testosterone stimulate the Sertoli cells, which together provide support for spermatogenesis.

Gonadal dysfunction may be classified as primary and/or secondary hypogonadism. Primary hypogonadism is due to testicular damage resulting in impaired spermatogenesis and/or dysfunction of the Leydig cells. As a rule of thumb, elevated FSH indicates impaired spermatogenesis, whereas permanently elevated LH indicates Leydig cell dysfunction. Secondary hypogonadism is caused by damage to the pituitary gland or hypothalamus, that is, after cranial radiotherapy, and leads to decreased LH and FSH followed by both exocrine and endocrine gonadal failure. Male gonadal function can thus be screened by assessment of serum levels of LH, FSH, testosterone, SHBG and sperm cell count and by the consideration of fatherhood. In case of Leydig cell damage the serum levels of testosterone are low and those of LH are often elevated, though Greenfield *et al* (2007) recently has cast some doubt about this correlation.

With this background, the aim of the present cross-sectional study was to assess post-treatment gonadal function in a large series of lymphoma patients in relation to treatment, age and observation time emphasizing the possible differences between HLSs and NHLSs.

## Patients and methods

### Patients

Eligible patients for the present postal cross-sectional study fulfilled all the following criteria: 1: male patients ⩽50 years at diagnosis, 2: treated for Hodgkin's lymphoma (HL) and/or non-Hodgkin's lymphoma (NHL), 3: registered in the lymphoma database at the Norwegian Radium Hospital (NRH), 4: Period of diagnosis from 1 January 1980 to 31 December 2002, 5: age above 18 years at the time of the survey (January 2007), 6: valid postal address and 7: alive in June 2007.

Patients who had received total brain irradiation or scrotal irradiation were excluded. Patients registered with both HL and NHL was grouped according to their first diagnosis. Totally there were 16 patients, of whom seven had their gonadal hormones assessed. Of these seven, four were initially diagnosed with HL and three with NHL, and later on they were diagnosed with a secondary lymphoma.

### Treatment

Treatment strategies of HL and NHL at the NRH for the period 1980–2002 have been described earlier (Hagemeister *et al*, 1987; Merk *et al*, 1991; Evensen *et al*, 1994; Abrahamsen *et al*, 1996; Holte *et al*, 1996; Hollender *et al*, 2000; Aurlien *et al*, 2001; Seidemann *et al*, 2001; Sweetenham *et al*, 2001; Blystad *et al*, 2001a, 2001b; Smeland *et al*, 2004; Kiserud *et al*, 2007).

For the purpose of this study the total treatment of each patient, thus both primary and eventual salvage therapy, was summarised as recorded in the lymphoma database. As there is little knowledge concerning damage of the Leydig cell function by chemotherapy, we have categorised chemotherapy according to the expected exocrine gonadal damage. Categories were created based mainly on literature on post-treatment exocrine hypogonadism, as reported in a previous study on post-treatment parenthood in HLSs: low, medium and high chemotherapy-induced gonadotoxicity (Viviani *et al*, 1985; Howell and Shalet, 2001; Lee *et al*, 2006; Kiserud *et al*, 2007). In addition, a group was constructed including patients who had received radiotherapy only.

Analyses showed no differences with respect to gonadal hormones between patients who had received supradiaphragmatic RT only and those who had been irradiated with inverted Y/inguinal or other infradiaphragmatic fields. In addition, there was no difference between the group treated with radiotherapy only and the group treated with low gonadotoxic chemotherapy.

The treatment groups were defined as follows: 1: radiotherapy only and/or low gonadotoxic chemotherapy (both HL and NHL) (‘No/low’), 2: medium gonadotoxicity chemotherapy for NHL (‘med-NHL’), 3: medium gonadotoxicity chemotherapy for HL (‘med-HL’), 4: highly gonadotoxic chemotherapy for NHL (‘high-NHL’), 5: highly gonadotoxic chemotherapy for HL (‘high-HL’). The different chemotherapy regimens were allocated to groups 1–5 as described in Table 1A, with chemotherapy regimens, number of courses and type of radiotherapy for each treatment group outlined in Tables 1B and 1C.

### Study design

The cross-sectional study included serum hormone analyses and a questionnaire assessing various aspects of quality of life. The present analyses are restricted to gonadal hormones, whereas quality of life aspects will be discussed in detail in a separate paper.

Blood samples for analyses of gonadal hormones (testosterone, SHBG, LH, FSH) were to be drawn before noon by the patient's GP and mailed to the Hospital's Department of Medical Biochemistry. Patients with gonadal hormones indicating hypogonadism were invited to an outpatient clinical examination and a control morning blood sample. All analyses were performed at the same laboratory at Rikshospitalet with E170 module for Modular Analytics, Roche Professional Diagnostic, Germany. Normal ranges were: Testosterone: 9.0–31.0 nmol l^−1^, SHBG: 15–85 nmol l^−1^, FSH<12.0 U l^−1^, LH<10.0 U l^−1^. The testosterone/SHBG ratio was calculated for each patient, as a surrogate for free testosterone, with testosterone/SHBG ratio <0.25 indicating hypogonadism.

The patients’ gonadal hormone levels were categorised into three groups: 1: All gonadal hormones within normal range (Normal), 2: Isolated elevated FSH, with LH, SHBG and testosterone within normal range (exocrine hypogonadism), 3: Testosterone below and/or LH above normal range, independent of FSH (endocrine hypogonadism), admitting that this group includes some male patients with both endocrine and exocrine hypogonadism. (Table 2).

Patients with endocrine hypogonadism were further sub-grouped: A: low testosterone and normal LH/FSH, B: elevated LH, testosterone normal, C: low testosterone combined with either elevated LH and/or elevated FSH. (Table 3).

Three patients already on testosterone substitution were included in subgroup C, assuming they had low testosterone combined with elevated LH before having started testosterone substitution.

### Ethical considerations

Informed written consent was obtained from all responders. The Regional Committee for Medical Research Ethics, Health Region South, Norway and the Institutional Review Board and the Chief Privacy Officer at the NRH approved the study.

### Statistical analyses

Data were described with medians and ranges. Continuous variables were compared using Mann–Whitney Wilcoxon and Kruskal–Wallis tests. Crude associations between categorical variables were computed with χ^2^ tests.

Age at diagnosis was categorised into four groups: 1: 6–25 years, 2: 26–34 years, 3: 35–41 years, 4: 42–50 years. Age at survey (January 2007) was categorised as follows: 1: 21–39 years, 2: 40–49 years, 3: 50–56 years, 4: 57–75 years. Observation time was calculated from date of first diagnosis to 1 January 2007, and was further categorised in three groups: 1: 4–10 years, 2: 11–20 years, 3: 21–28 years.

Multinomial regression analyses were performed, the dependent variable being gonadal hormones divided into three categories: (1=normal (reference), 2=exocrine hypogonadism, 3=endocrine hypogonadism). The model was adjusted for the five treatment groups, age groups and observation time. As initial analyses revealed that observation time was not significant, this variable was excluded from the final model. Finally, two multivariable models were fitted; one including treatment groups and age at survey as independent variables, the other included treatment groups and age at diagnosis. The odds ratios for both exocrine and endocrine hypogonadism were similar in both models, possibly due to a strong correlation between age at diagnosis and age at survey. Only results from the model fitted with treatment and age at survey are presented.

*P*<0.05 were considered statistically significant (all tests were two-sided). All analyses were performed using SPSS 13 for Windows.

## Results

### Patients’ characteristics

In all 653 patients met the inclusion criteria and were invited to participate by mail (360 HL, 293 NHL). A total of 294 (45%) had their gonadal hormones assessed and were included in this study (compliers).

The compliers and non-compliers (survivors without blood samples) were similar as to lymphoma diagnosis and treatment groups. However, the compliers were older than the non-compliers both at diagnosis (median 33 *vs* 31 years (*P*=0.006)) and at survey (median 49 *vs* 45 years (*P*<0.001)), and had significantly longer observation time (median 15 *vs* 13 years (*P*=0.028)).

Of the 294 compliers, 165 (56%) had been treated for HL and 129 (44%) for NHL. Sixty-four percent (*n*=187) had received chemotherapy and radiotherapy, 63 (21%) had had chemotherapy only and 44 patients (15%) had undergone radiotherapy only. Median age at diagnosis was 33 years (range, 6–49), median age at survey was 49 years (range, 21–73), and median observation time was 15 years (range, 4–28) (Table 2).

As expected, age at survey and age at diagnosis were higher in NHLSs compared with HLSs, without difference in observation time between these groups.

### Single values of FSH, LH, Testosterone and SHBG

The median FSH value for all patients was 8.5 U l^−1^ (range, 0.4–67.0 U l^−1^). Forty-one percent had FSH values > 12.0 U l^−1^, and in 26% the FSH value were > 20.0 U l^−1^. The median LH value was 6.0 U l^−1^ (range, 1.2–45.8 U l^−1^), and 16% of the patients had elevated LH (>10.0 U l^−1^) (Table 4).

The median testosterone value was 12.9 nmol l^−1^ (range, 1.7–33.9 nmol l^−1^), with 20% of the patients having testosterone value <9.0 nmol l^−1^. The median value of the testosterone/SHBG-ratio was 0.37. Of all patients, 14% had a testosterone/SHBG ratio <0.25.

There was a significant association between age at survey and the individual levels of FSH, LH and SHBG (*P*<0.001). For testosterone, no such association with age at survey was observed. However, the testosterone/SHBG-ratio decreased significantly with increasing age (*P*<0.001).

There was also a significant association between the individual levels of both FSH, LH (*P*<0.001) and SHBG (*P*=0.042) and treatment groups. The median level of FSH and LH increased significantly in all other treatment groups compared with patients with no/low gonadotoxic chemotherapy (*P*<0.001–*P*<0.02). For testosterone and testosterone/SHBG-ratio no association with treatment groups was found.

### Groups of gonadal dysfunction

**Figure 1:**
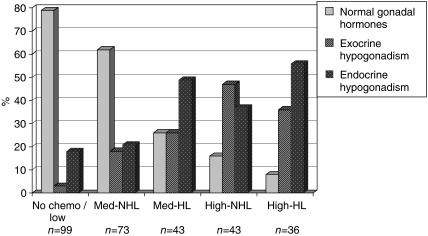
Proportions of male lymphoma survivors with normal gonadal hormones, exocrine and endocrine hypogonadism. Normal gonadal hormones: testosterone, SHBG, LH and FSH within normal ranges. Exocrine hypogonadism: isolated elevated FSH. Testosterone, SHBG, LH, within normal ranges. Endocrine hyogonadism: elevated LH and/or low testosterone. Treatment groups: No chemo/low: radiotherapy only/low gonadotoxic chemotherapy, Med-NHL: medium gonadotoxic chemotherapy for non-Hodgkin's lymphoma (NHL), Med-HL: medium gonadotoxic chemotherapy for Hodgkin's lymphoma (HL), High-NHL: highly gonadotoxic chemotherapy for NHL, High-HL: highly gonadotoxic chemotherapy for HL.

Forty-nine percent (*n*=144) had all gonadal hormones within normal ranges, 60 (20%) displayed exocrine hypogonadism (isolated elevated FSH, with LH, SHBG and testosterone within normal range) and in almost one-third (*n*=90, 30%) the hormone levels were compatible with endocrine hypogonadism (testosterone below and/or LH above normal range, independent of FSH). There was a statistically significant association between treatment group and groups of gonadal dysfunction (*P*<0.001). After treatment with no/low gonadotoxic chemotherapy 79% had normal gonadal hormones, whereas the comparable figures were 62% after med-NHL, 26% after med-HL, 16% after high-NHL and 8% after high-HL (Figure 1; (Table 2).

Of the 41 patients treated with high dose chemotherapy and autologous stem cell support (HDT), 19 men had exocrine and 20 endocrine hypogonadism. Only two patients had normal gonadal hormones.

Stage at diagnosis was significantly associated with the group of hormonal dysfunction (*P*<0.001). In patients with stage I/II at diagnosis 62% had normal gonadal hormones, 11% displayed exocrine hypogonadism and endocrine hypogonadism was demonstrated in 27%. The comparable numbers in patients with stage III/IV were: normal gonadal hormones in 30%, exocrine hypogonadism in 34% and endocrine hypogonadism in 36%.

The patients with endocrine hypogonadism were older both at diagnosis (*P*=0.004) and at survey (*P*<0.001) and they had a longer median observation time (*P*=0.047) than those with normal gonadal hormones. There was no difference in age at diagnosis or survey and in observation times between patients with exocrine hypogonadism and patients with normal gonadal hormones.

### Multinomial regression analyses

Compared to those treated with no/low gonadotoxic chemotherapy patients from all other treatment groups had significantly elevated odds ratios (OR) for exocrine hypogonadism (Table 5). Patients in the med-NHL group were 6.3 (95% CI: 1.7–23.8) times more likely to suffer from exocrine hypogonadism compared to those treated with no/low gonadotoxic chemotherapy. The comparable ORs for the other groups were 25.7 (95% CI: 6.2–107.0) after med-HL, 73.3 (95% CI: 17.2–312.2) after high-NHL and 112.0 (95% CI: 20.1–625.2) after high-HL. Age at survey was not significantly associated with exocrine hypogonadism.

In addition, compared to the group treated with no/low gonadotoxic chemotherapy, patients from the other treatment groups, except those in the med-NHL group, had significantly elevated risk also for endocrine hypogonadism. Patients in the med-HL were 8.0 (95% CI: 3.2–20.4) times more likely to have endocrine hypogonadism compared to those treated with no/low gonadotoxic chemotherapy, whereas the comparable ORs for high-NHL were 10.5 (95% CI: 3.6–30.6) and 37.2 (95% CI: 9.4–147.7) for high-HL.

Men aged above 50 years at survey were about five times more likely to have endocrine hypogonadism compared with those less than 40 years at survey.

The ORs for both isolated elevated FSH and endocrine hypogonadism increased more in HL survivors compared with NHL survivors in comparable treatment groups.

Overall, treatment with medium and highly gonadotoxic chemotherapy for both HL and NHL had a stronger effect on exocrine than on endocrine hypogonadism.

### Patients with endocrine hypogonadism (*n*=90)

Patients with low testosterone combined with elevated LH and/or FSH displayed significantly longer observation times than the other two subgroups (*P*=0.001). This group had also significantly lower testosterone/SHBG ratios than the other subgroups (*P*=0.003/*P*=0.001). (Table 3).

## Discussion

In this cross-sectional observational study gonadal hormones were assessed in 294 male survivors after treatment for both HL and NHL. About half had all gonadal hormones within normal range, 20% had isolated elevated FSH, and almost one-third had gonadal hormones compatible with our definition of endocrine hypogonadism. Compared to those treated with no/low gonadotoxic chemotherapy, patients from all other treatment groups had significantly elevated risk for exocrine hypogonadism. In addition, patients from the other treatment groups, except those in the med-NHL group, had a significantly elevated risk also for endocrine hypogonadism compared to the group treated with no/low gonadotoxic chemotherapy.

We confirm the results from our previous study in HLSs, where we observed exocrine gonadal damage related to the type, cumulative dose and intensity of chemotherapy, assessed by post-treatment paternity (Kiserud *et al*, 2007). This study shows for the first time that the same categorization of chemotherapy in low, medium and highly gonadotoxic treatment can be useful also when exploring endocrine hypogonadism.

Forty-one percent of our lymphoma survivors had elevated FSH. Although this figure seems comparable to 35%, reported by van der Kaaij *et al* (2007), important differences should not be overseen. Our series included men with initially advanced disease and men treated for relapse. Many of our patients thus have had more intensive chemotherapy than usually given to patients with stage I/II. In addition, we examined both HLSs and NHLSs, which make the two series less comparable. Finally, our median observation time was 15 years, compared with the median 32 months follow-up period reported by van der Kaaij *et al* (2007). We thus consider our proportion of FSH elevation as definite, whereas one still can expect some recovery of spermatogenesis with longer follow up in early stage lymphoma patients with limited treatment.

This study confirms the low risk of elevated FSH in HLSs after treatment with ABVD-like regimens or radiotherapy only compared with other combinations incorporating alkylating agents (Whitehead *et al*, 1982; Viviani *et al*, 1985, 1991; van der Kaaij *et al*, 2007). In addition, we observed that the probability of exocrine hypogonadism increased with treatment intensity also in NHLSs, whereas age at survey was of no significance.

Compared to the no/low gonadotoxic chemotherapy group the risk of endocrine hypogonadism increased for all treatment groups except for NHLSs treated mainly with CHOP-based chemotherapy (med-NHL). Intensification of chemotherapy increased the risk of endocrine gonadal failure more in HLSs than in NHLSs. This probably reflects that chemotherapy regimens used for HL generally are more gonadotoxic than those used for NHL. This difference may also be due to differences in the pre-treatment gonadal function between HLSs and NHLSs, pointed out by Botchan *et al* (1997). Increasing risk of endocrine hypogonadism along with chemotherapy intensification has also been documented for testicular cancer survivors (Nord *et al*, 2003).

In this paper, we followed the categorisation for endocrine hypogonadism based on elevated LH and normal testosterone (compensated hypogonadism) or low testosterone with or without elevated LH proposed by Howell *et al* (1999). Our data indicated that the duration of the observation time might be associated with the development of an uncompensated hypogonadism, as this period was longer in subgroup 3C than in subgroup 3A and 3B. However, our patient sample also shows a more diverse picture, with 27 of 57 patients with low testosterone having normal LH (subgroup 3A). Similarly, Greenfield *et al* (2007) reported low testosterone values (<10 nmol l^−1^) in 24 (13.6%) of 176 patients, but only eight of these men had increased LH. This may reflect that causes of endocrine hypogonadism in cancer survivors may be more complex than previously described. Chemotherapy and/or radiation-induced damage may develop either in the gonads, the hypothalamus–pituitary axis or as a combination of these two. To verify the influence of secondary hypogonadism, the pituitary function has to be tested by a gonadotropin-releasing hormone test, which was not performed in our patients. Further, except for the pathway through the hypothalamus–pituitary axis and the release of GnRH, the mechanisms related to Leydig cell dysfunction after cancer treatment is not well characterised. The possible negative influence of germinative dysfunction on Leydig cells suggested by Howell *et al* (1999) is thus interesting, but requires further examination.

The blood samples were generally taken during the morning. We were aware of the risk of falsely low testosterone in case of afternoon blood sampling and tried to avoid this possibility as much as possible. However, we completely cannot exclude such sampling as explanation in some patients in subgroup 3A. For most of these patients, the results have been confirmed in a subsequent morning sample taken at the hospital's outpatient clinic, as patients with gonadal hormones indicating hypogonadism were invited to clinical examination and a control morning blood sample. Finally, a low testosterone value with normal LH and FSH may also be due to a random low testosterone value caused by, for instance patients’ stress.

Testosterone deficiency in adult males has been associated with declining bone mass, muscle strength, reduced energy levels and altered sexual function (Araujo *et al*, 2007). In male cancer survivors, Howell *et al* (2000) reported reduced bone mineral density in patients with mild Leydig cell impairment, and testosterone levels has been reported to be negatively correlated to insulin, total body fat mass/total trunk fat mass and BMI (Huddart and Norman, 2003; Greenfield *et al*, 2007). With this background, it is of clinical importance to identify male cancer survivors with endocrine hypogonadism. In this context, it is surprising that only three of the included patients reported testosterone substitution therapy at the time of the survey, whereas endocrine hypogonadism was found in one-third of our patients.

Testosterone replacement therapy is a challenging issue in both male cancer survivors and in the general male population. Age has a significant influence on physiological testosterone levels. To evaluate endocrine hypogonadism at least two morning blood samples are needed and clinical symptoms, age and possible contraindications for testosterone substitution have to be considered. Owing to these caveats our study patients’ with endocrine hypogonadism identified in this study were offered repeated hormone analyses and clinical examination at the outpatient clinic before any testosterone substitution was initiated.

There are some limitations to be discussed:

The compliers were older at survey than the non-compliers. This may have caused a biased patient sample with a slightly higher proportion of hypogonadism in our patient sample than in the total population of male lymphoma survivors.

Our patients’ age ranged from 21 to 73 years at survey and we found a fivefold increase in the risk of endocrine hypogonadism in men aged above 50 years at survey compared to those less than 40 years at survey. Similarly, Howell *et al* (1999) reported increasing risk of chemotherapy-induced Leydig cell damage with increasing age, and an age-dependent decline in testosterone levels was reported by Greenfield *et al* (2007). Studies from the general population have shown age-related decrease in testosterone values (Svartberg *et al*, 2003; Mohr *et al*, 2005). As our normal range for testosterone was not age adjusted, this may have lead to a comparatively broad allocation of older men to the group of endocrine hypogonadism. As a further limitation we could not consider chronic co-morbidity or any long-lasting medication in our patients, which might also have influenced our findings.

Seven patients registered in the database with both HL and NHL had their gonadal hormones assessed, and were grouped according to their first diagnosis. This may have biased the results because their treatment consisted of combinations of different types of chemotherapy.

The strength of this cross-sectional study is that it consists of a large series of male survivors after both HL and NHL with known treatment details and gonadal hormones assessed by a single laboratory. We achieved a compliance of 45%, which we consider satisfactory taken into account that this long-term follow-up survey implied blood sampling at a GP's office.

The results of this study implicate that gonadal hormones should be assessed regularly in male lymphoma survivors, especially after treatment with high-dose alkylating agents and HDT and in male patients 50 years and older. If gonadal hormones indicate hypogonadism, further analyses and clinical evaluation should evaluate the need of testosterone substitution to avoid the development of serious health problems.

## Figures and Tables

**Table 1A:** Groups of chemotherapy according to expected gonadotoxicity

**Treatment groups**	**Treatment**
1. No chemotherapy/ Low gonadotoxic chemotherapy (both HL and NHL)	Radiotherapy only ABVD/EBVP and similar
No/low	
2. Medium gonadotoxic chemotherapy NHL	CHOP/COP/CHOEP ⩽8 courses alone
	CHOP ⩽8 courses combined with high dose Mtx
Med-NHL	MACOP B
	BFM 90/93
	Chlorambucil p.o.
	MIME
3. Medium gonadotoxic chemotherapy HL	LVPP ⩽4 courses alone or combined with ABOD/EBVP/dexa BEAM ( 2 courses)
	OEPA+0–4 COPP
	MIME
Med-HL	
4. Highly gonadotoxic chemotherapy NHL	CHOP > 8 courses
	CHOP=8 courses combined with MIME, ENAP or Chlorambucil
	Maxi-CHOP ⩾6 courses
High-NHL	HDT with BEAM as conditioning regimen
	HDT with cyclophosphamide and TBI as conditioning regimen
5. Highly gonadotoxic chemotherapy HL	LVPP > 4 courses
	LVPP=4 courses combined with CHOP, MIME or BEACOPP (4 courses)
	BEACOPP 8 courses
High-HL	HDT with BEAM as conditioning regimen
	HDT with cyclophosphamide and TBI as conditioning regimen

HDT=high-dose chemotherapy with autologous stem cell support; TBI=total body irradiation.

**Table 1B:** Chemotherapy regimens with cumulative doses of alkylating agents and procarbazine used in the five treatment groups

	**1.No chemo/low**	**2. Med-NHL**	**3. Med-HL**	**4. High-NHL**	**5. High-HL**
	***N*=99**	***N*=73**	***N*=43**	***N*=43**	***N*=36**
	**No. of patients (%)**	**No. of patients (%)**	**No. of patients (%)**	**No. of patients (%)**	**No. of patients (%)**
Chemotherapy only	10 (10)	30 (41)	6 (14)	7 (16)	10 (28)
Radiotherapy only	42 (42)				
Chemotherapy+radiotherapy	47 (48)	43 (59)	37 (86)	36 (84)	26 (72)
1st relapse	6	8	11	24	13
⩾2nd relapse	1	1	2	10	3
ABVD/EBVP ⩽8 courses Doxorubicin ⩽400 mg m^−2^	57	1	29	1	13
					
OEPA ⩽2 courses			3		
Doxorubicin ⩽160 mg m^−2^					
					
LVPP ⩽4 courses			38	1	6
Chlorambucil ⩽336 mg m^−2^					
Procarbazine ⩽5.6 g m^−2^					
					
LVPP 6–8 courses					18
Chlorambucil 504–672 mg m^−2^					
Procarbazine 8.4–11.2 g m^−2^					
					
BEACOPP					5
8 standard courses or					
2 dose escalated/6 standard					
Cyclophosphamide 5.2–6.4 g/m^2^					
Procarbazine 5.6 g m^−2^					
					
BEACOPP					1
4 standard courses					
Cyclophosphamide 2.6 g m^−2^					
Procarbazine 2.8 g m^−2^					
					
COPP ⩽2 courses			2		
Cyclophosphamide ⩽1 g m^−2^					
Procarbazine ⩽3 g m^−2^					
					
CHOP/COP ⩽8 courses		58		23	4
Cyclophosphamide ⩽6 g m^−2^					
					
CHOP/COP >8 courses				8	1
Maxi-CHOP ⩾6 courses					
Cyclophosphamide ⩾6.75 g m^−2^					
					
MACOP B		6			
Doxorubicin 300 mg m^−2^					
Cyclophosphamide 2.1 g m^−2^					
					
BFM 90/93		3			
Cyclophosphamide 3 g m^−2^					
Ifosfamide 12 g m^−2^					
					
MIME ⩽4 courses			1	20	9
Ifosfamide ⩽20 g m^−2^					
					
MIME 5–7 courses		2		5	3
Ifosfamide 25–35 g m^−2^					
					
Chlorambucile p.o.		7		8	
1080 mg/6 months					
					
HDT/BEAM				13	8
Melphalan 140 mg m^−2^					
Carmustin 300 mg m^−2^					
					
TBI/cyclophoshamide				18	2
Cyclophosphamide					
60 mg kg^−1^ × 2					

ABVD=doxorubicin, bleomycin, vinblastine, dacarbazine; BEACOPP=cyclophosphamide, doxorubicin, etoposide, procarbazine, prednisone, vincristine, bleomycine; BEAM=carmustine, etoposide, cytarabine, melphalan; CHOP=cyclophosphamide, doxorubicin, vincristine, prednisone; LVPP, chlorambucile, vinblastine, procarbazine, prednisone; MACOP-B, methotrexate, doxorubicin, cyclophosphamide, vincristine, prednisone, bleomycin; MIME, mitoguazone, iphosphamide, methotrexate, etoposide; OEPA, doxorubicine, vincristine, etoposide, prednisone.

Number of patients=294.

BFM 90/93 as described by Seidemann *et al*, 2001, see reference list.

**Table 1C:** Radiotherapy treatment in the five treatment groups

	**1. No chemo/low**	**2. Med-NHL**	**3. Med-HL**	**4. High-NHL**	**5. High-HL**
	***N*=99**	***N*=73**	***N*=43**	***N*=43**	***N*=36**
	**(%)**	**(%)**	**(%)**	**(%)**	**(%)**
Received radiotherapy	89 (90)	43 (59)	37 (86)	36 (84)	26 (72)
Supradiaphragmatic	73	22	24	11	15
Abdominal fields	3	16	3	3	4
InvertedY/Inguinal fields	10	4	9	3	5
TBI				18	2
Other radiation fields	3	1	1	1	

Number of patients=294.

HL:

Stage I/II:

1980–1982: 2 Gy × 20, 1982–1998: 1.8 Gy × 23, 1999 onwards: 1.75 Gy × 17.

Stage III/IV:

1980–1985: 2 Gy × 20, 1985–1998: 2 Gy × 20 or 1.8 Gy × 23, 1999 onwards: 1.75 Gy × 17.

NHL:

Low-grade: 2Gy × 15 to involved and nearest neighbouring draining lymph node region.

High-grade: 2 Gy × 20 or 1.8 Gy × 23 if residual masses or initial Bulky disease.

TBI (total body irradiation): 1.3 Gy × 10.

**Table 2:** Patients characteristics

	**All**	**Normal gonadal hormones**	**Exocrine hypogonadism**	**Endocrine hypogonadism**
	***N*=294**	***N*=144**	***N*=60**	***N*=90**
		**(49%)**	**(20%)**	**(31%)**
Median (range) age at diagnosis^a^	33 (6–49)	31 (6–49)	33 (11–48)	35 (14–49)
Median (range) age at survey^b^	49 (21–73)	47 (21–68)	47 (25–67)	54 (28–73)
Median (range) observation time^c^ (years)	15 (4–28)	14 (4–26)	16 (4–28)	17 (4–26)
				
	**No. of patients (% in this group)**	**No. of patients (% in this group)**	**No. of patients (% in this group)**	**No. of patients (% in this group)**
HL	165 (56)	79 (55)	27 (45)	59 (66)
Stage I/II^d^	105	62	12	31
Stage III/IV^d^	60	17	15	28
First relapse	27	5	8	14
⩾2nd relapse	5			5
NHL	129 (44)	65 (45)	33 (55)	31 (34)
Stage I/II^d^	67	45	7	15
Stage III/IV^d^	62	20	26	16
First relapse	35	8	17	10
⩾2nd relapse	12	2	8	2
HL/NHL	7	3	1	3
				
**Treatment group**	**No. of patients (% in this group)**	**No. of patients**	**No. of patients**	**No. of patients**
1. No chemo/Low	99 (34)	78	3	18
2. Med-NHL	73 (25)	45	13	15
3. Med-HL	43 (15)	11	11	21
				
4. High-NHL	43 (15)	7	20	16
HDT/BEAM	13	1	8	4
TBI/cyclophoshamide	18	1	7	10
				
5. High-HL	36 (12)	3	13	20
HDT/BEAM	8		4	4
TBI/cyclophoshamide	2			2

Exocrine hypogonadism: FSH>12.0 U l^−1^; testosterone, SHBG, LH within normal ranges.

Endocrine hypogonadism: Either testosterone <9.0 nmol l^−1^ and/or elevated LH.

Treatment groups:

**1:** No/Low gonadotoxic chemotherapy±radiotherapy.

2: Medium gonadotoxic chemotherapy±radiotherapy NHL.

3: Medium gonadotoxic chemotherapy±radiotherapy HL.

4: High gonadotoxic chemotherapy±radiotherapy NHL.

5: High gonadotoxic chemotherapy±radiotherapy HL.

a Age at diagnosis: *P*=0.017, Kruskal–Wallis (among groups of gonadal hormones).

b Age at survey: *P*=0.001, Kruskal–Wallis (among groups of gonadal hormones).

c Observation time: time from diagnosis to 1 January 2007 (in years).

d Stage at initial diagnosis.

HDT=high dose chemotherapy with autologous stem cell support.

HL/NHL=patients registered with both diagnosis in the lymphoma database.

**Table 3:** Characteristics of the patients with endocrine hypogonadism, and subgroups

		**A.**	**B.**	**C.**
	**Endocrine**	**Low testosterone**	**Elevated LH**	**Low testosterone**
	**hypogonadism**	**LH/FSH normal**	**Testosterone normal**	**Elevated LH or FSH or both^a^**
	***N*=90**	***N*=27**	***N*=30**	***N*=33**
Median (range) age at diagnosis	35 (14–49)	33 (18–46)	40 (18–49)	33 (14–49)
Median (range) age at survey	54 (28–73)	48 (28–59)	54 (29–68)	57 (33–73)
Median (range) observation time	17 (4–26)	14 (4–25)	16 (4–26)	20 (4–26)
Median (range) Testosterone/SHBG ratio	0.27 (0.05–0.85)	0.33 (0.13–0.49)	0.34 (0.17–0.72)	0.23 (0.05–0.85)
				
Diagnosis	% within this group			
HL	59 (66)	22	15	22
NHL	31 (34)	5	15	11
				
*Treatment group*				
1. No chemo/Low	18	15	2	1
2. Med-NHL	15	4	5	6
3. Med-HL	21	3	6	12
				
4. High-NHL	16	1	10	5
HDT/BEAM	4		4	
TBI/cyclophosphamide	10	1	4	5
				
5. High-HL	20	4	7	9
HDT/BEAM	4	1	3	
TBI/cyclophosphamide	2	1		1

Treatment groups:

1: No/Low gonadotoxic chemotherapy±radiotherapy.

2: Medium gonadotoxic chemotherapy±radiotherapy NHL.

3: Medium gonadotoxic chemotherapy±radiotherapy HL.

4: High gonadotoxic chemotherapy±radiotherapy NHL.

5: High gonadotoxic chemotherapy±radiotherapy HL.

a Elevated FSH and low testosterone: *n*=17, elevated both LH and FSH and low tesosterone *n*=16.

**Table 4:** Serum levels of LH, FSH, testosterone, SHBG and testosterone/SHBG ratio related to age at survey and treatment groups

		**Age at survey groups**				**Treatment groups**				
										
		**1**	**2**	**3**	**4**	**1**	**2**	**3**	**4**	**5**
	**All**	**21–39 yrs**	**40–49 yrs**	**50–56 yrs**	**57–75 yrs**	**No/low**	**med-NHL**	**med-HL**	**high-NHL**	**high-HL**
	***N*=294**	***N*=66**	***N*=86**	***N*=75**	***N*=67**	***N*=99**	***N*=73**	***N*=43**	***N*=43**	***n*=36**
*FSH*										
Median	8.5	5.9	6.4	8.5	17.7	4.7	7.2	19.8	19.2	20.5
Range (U l^−1^)	0.4–67.0	1.0–32.0	1.4–67.0	2.3–56.4	0.4–63.3	1.0–40.4	1.4–41	2.2–56.4	2.3–67.0	0.4–53.2
FSH >12 U l^−1^	41%	31%	32%	43%	59%	5%	33%	70%	81%	81%
										
*LH*										
Median	6.0	5.7	5.5	5.7	7.7	4.4	5.4	7.9	8.2	8.2
Range U l^−1^	1.2–45.8	1.8–14.5	1.7–20.1	1.8–26.3	1.2–45.8	1.7–16.7	1.2–43.6	1.9–19.6	2.3–45.8	4.5–20.1
LH >10 U l^−1^	16%	10%	7%	23%	25%	3%	12%	30%	29%	32%
										
*Testosterone*										
Median	12.9	13.6	13.7	12.8	11.1	12.6	14.3	10.2	14.2	11.3
Range (nmol l^−1^)	1.7–33.9	4.4–28.1	4.7–33.9	4.3–26.6	1.7–23.0	5.2–27.1	1.7–30.4	5.4–33.9	4.3–22.4	4.4–29.6
Testosterone <9 nmol l^−1^	20%	12%	16%	24%	28%	17%	14%	33%	15%	33%
										
*SHBG*										
Median	34	26	32	41	42	33	40	31	32	29
Range (nmol l^−1^)	10–132	10–84	11–71	17–84	14–132	10–84	13–132	16–70	14–66	14–76
										
*Testosterone/SHBG ratio*										
Median	0.37	0.49	0.42	0.32	0.28	0.42	0.35	0.31	0.36	0.39
Range	0.05–1.02	0.18–1.02	0.13–0.85	0.14–0.65	0.05–0.56	0.13–0.78	0.05–0.84	0.14–1.02	0.1–0.71	0.1–0.85
Testosterone/SHBG ratio <0.25	14%	3%	9%	15%	30%	13%	10%	24%	15%	14%

Age at survey categorised in four groups: 1: 21–39 years, 2: 40–49 years, 3: 50–56 years, 4: 57–75 years.

Treatment groups:

1: No/Low gonadotoxic chemotherapy±radiotherapy.

2: Medium gonadotoxic chemotherapy±radiotherapy NHL.

3: Medium gonadotoxic chemotherapy±radiotherapy HL.

4: High gonadotoxic chemotherapy±radiotherapy NHL.

5: High gonadotoxic chemotherapy±radiotherapy HL.

**Table 5:** Odds for exocrine and endocrine hypogonadism

	**OR**	**95% CI**	***P*-value**
*Exocrine hypogonadism*			
*Treatment*			
1.No/low	Reference		
2.Med-NHL	6.3	1.7–23.8	0.007
3.Med-HL	25.7	6.2–107.0	<0.001
4.High-NHL	73.3	17.2–312.2	<0.001
5.High-HL	112.0	20.1–625.2	<0.001
			
* Age at survey*			
1: 21–39 years	Reference		
2: 40–49 years	1.0	0.4–2.7	0.96
3: 50–56 years	1.0	0.3–3.0	0.99
4: 57–75 years	2.0	0.7–5.8	0.19
			
*Endocrine hypogonadism*			
* Treatment*			
1.No/low	Reference		
2.Med-NHL	1.1	0.5–2.5	0.86
3.Med-HL	8.0	3.2–20.4	<0.001
4.High-NHL	10.5	3.6–30.6	<0.001
5.High-HL	37.2	9.4–147.7	<0.001
			
* Age at survey*			
1: 21–39 years	Reference		
2: 40–49 years	1.5	0.5–3.9	0.46
3: 50–56 years	5.0	1.9–13.2	0.001
4: 57–75 years	4.9	1.8–13.7	0.002

Multinomial regression analysis.

Treatment groups:

1: No/Low gonadotoxic chemotherapy±radiotherapy.

2: Medium gonadotoxic chemotherapy±radiotherapy NHL.

3: Medium gonadotoxic chemotherapy±radiotherapy HL.

4: High gonadotoxic chemotherapy±radiotherapy NHL.

5: High gonadotoxic chemotherapy±radiotherapy HL.
